# Operation of national coordinating service for interhospital transfer from emergency departments: experience and implications from Korea

**DOI:** 10.1186/s12873-023-00782-1

**Published:** 2023-02-10

**Authors:** Hye Sook Min, Ho Kyung Sung, Goeun Choi, Hyehyun Sung, Minhee Lee, Seong Jung Kim, Eunsil Ko

**Affiliations:** 1 Public Health Research Institute, National Medical Center, Seoul, South Korea; 2 National Emergency Medical Center, National Medical Center, 245 Eulgi-ro, Jung-gu, Seoul, 04564 South Korea; 3 Seoul National University College of Nursing, Seoul, South Korea; 4 Department of Nursing, Graduate School, Chosun University, Gwangju, South Korea; 5 Department of Emergency Medicine, College of Medicine, Chosun University, Gwangju, South Korea

**Keywords:** Interhospital transfer, Emergency department, Coordination, Protocolized process, Information system

## Abstract

**Background:**

Since 2014, Korea has been operating the National Emergency Medical Situation Room (NEMSR) to provide regional emergency departments (EDs) with coordination services for the interhospital transfer of critically ill patients. The present study aimed to describe the NEMSR’s experience and interhospital transfer pattern from EDs nationwide, and investigate the factors related to delayed transfers or transfers that could not be arranged by the NEMSR.

**Methods:**

This study was a retrospective cross-sectional analysis of the NEMSR’s coordination registry from 2017 to 2019. The demographic and hospital characteristics related to emergency transfers were analyzed with hierarchical logistic models.

**Results:**

The NEMSR received a total of 14,003 requests for the arrangement of the interhospital transfers of critically ill patients from 2017 to 2019. Of 10,222 requests included in the analysis, 8297 (81.17%) successful transfers were coordinated by the NEMSR. Transfers were requested mainly due to a shortage of medical staff (59.79%) and ICU beds (30.80%). Delayed transfers were significantly associated with insufficient hospital resources. The larger the bed capacity of the sending hospital, the more difficult it was to coordinate the transfer (odds ratio [OR] for transfer not arranged = 2.04; 95% confidence interval [CI]: 1.48–2.82, ≥ 1000 beds vs. < 300 beds) and the longer the transfer was delayed (OR for delays of more than 44 minutes = 2.08; 95% CI: 1.57–2.76, ≥ 1000 beds vs. < 300 beds).

**Conclusions:**

The operation of the NEMSR has clinical importance in that it could efficiently coordinate interhospital transfers through a protocolized process and resource information system. The coordination role is significant as information technology in emergency care develops while regional gaps in the distribution of medical resources widen.

**Supplementary Information:**

The online version contains supplementary material available at 10.1186/s12873-023-00782-1.

## Background

Interhospital transfer from emergency departments (EDs) is critical for providing acute care in a regionalized emergency care system. When emergency care needs exceed the capacity and capability of regional hospitals, patients should be transferred to a larger hospital within a reasonable distance. However, in remote areas where medical resources are scarce, difficulties and delays in interhospital transfers may occur because there is not enough expertise nearby [1]. Furthermore, the arrangement for an interhospital transfer of a critically ill patient is a daunting and complex task for sending hospitals [2]. It is quite burdensome to determine the destination without any preexisting interhospital relationship. The transfer coordinator should identify the resource availability of the referring hospital and negotiate the transfer [1–4]. In such a transfer process, a public coordinating service can offer practical assistance for an ED needing to transfer patients. For example, a protocolized and preemptive negotiation by a public coordinator can efficiently arrange the transfer process between distant hospitals [5].

Korea has been operating a national coordinating service for interhospital transfer from EDs since 2014 to assist EDs having difficulties in selecting and arranging referral hospitals [6]. When it is decided that an emergency patient needs to be transferred to another hospital for advanced treatment, the staff or coordinators of the ED try to arrange the transfer. However, if the destination cannot be determined despite several attempts to arrange the transfer, the ED can seek assistance from the National Emergency Medical Situation Room (NEMSR) in Korea. Utilizing a protocolized process, communication by a public coordinator, and the electronic resource availability system, the NEMSR has arranged many interhospital transfers and determined referral hospitals for the sending EDs.

Since the NEMSR coordinates interhospital transfers requested from EDs across the country, examining the NEMSR’s coordination registry can help to better understand the process and challenges of transferring patients between hospitals. The present study aimed to 1) describe the outcomes of the NEMSR’s operation and the main reason for transferring patients from sending hospitals, and 2) analyze the factors related to delayed transfers or transfers that could not be arranged by the NEMSR. This study is expected to provide knowledge for the operation and outcome of a national coordinating service for the interhospital transfer of critically ill patients. The detailed analysis of interhospital transfers could help identify policy targets for the effective provision of emergency care.

## Methods

### Setting and operating procedures of the NEMSR

The NEMSR was established at the National Emergency Medical Center of Korea in 2014 to facilitate emergency transfers between the EDs designated by the government. In Korea, around 400 EDs nationwide have been designated by central and local governments and classified into three levels (I, II, and III) according to hospital resources and capabilities (including facilities, equipment, and medical staff) [7, 8]. Level I EDs of the region are accessible within 60 minutes by ground transportation from each of the 29 regions across the country and can provide definite care for critically ill patients. Level II EDs and level III EDs aim to ensure access to an ED within 30 minutes that can provide initial resuscitation and stabilization in the ED (Supplementary Fig. 1). For more understanding of the distribution and utilization of EDs in Korea, the size and distribution of hospitals, the number of beds per population, and the occupancy rate of ED beds are presented in Supplementary Tables 1, 2, 3 and 4. As the interhospital transfer of critically ill patients occurs within a region or between regions, the NEMSR centrally coordinates interhospital transfers when EDs request assistance. In the NEMSR, more than one emergency physician and two or three coordinators routinely monitor emergency medical resources and arrange transfers in a 24/7 manner. The National Emergency Medical Resource Information System (NEMRIS) is an electronic information system that provides medical resource information to the NEMSR. It provides near real-time information on hospital resources nationwide, such as the availability of ventilators, extracorporeal membrane oxygenation machines, hemodialysis machines, intensive care units (ICUs), and operating rooms.

**Fig. 1:**
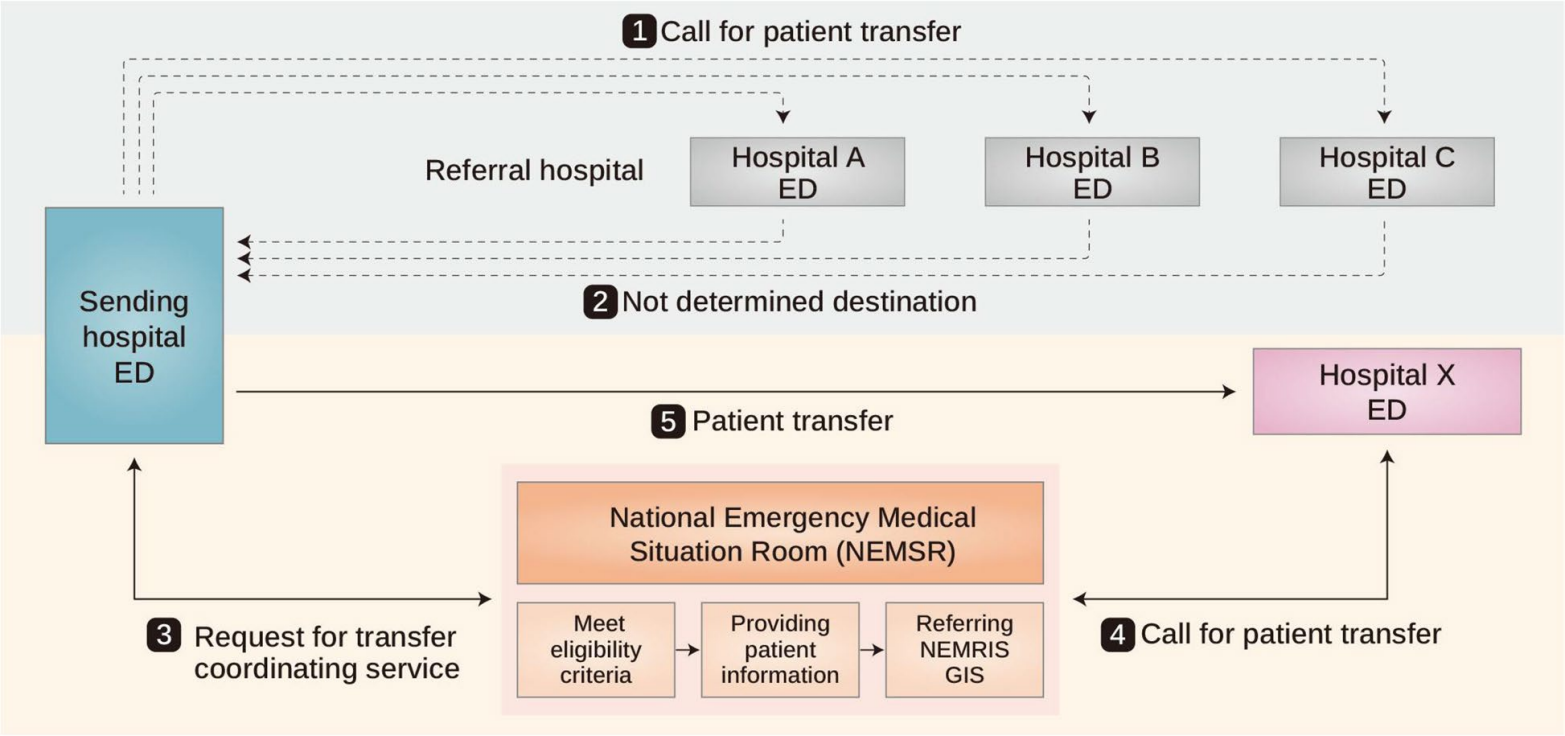
Transfer process coordinated by the National Emergency Medical Situation Room (NEMSR). NEMRIS, National Emergency Medical Resource Information System; GIS, geographic information system

Figure 1 shows the NEMSR’s operational procedure for interhospital transfer coordination. The eligibility criteria for NEMSR’s assistance are 1) transfers only from an ED, 2) the transfer of critically ill patients requiring emergency surgery, procedures, or other treatments who cannot receive them at the sending hospital, and 3) transfers requested by medical staff in the ED. The transfer request is not eligible for coordination if the patient does not want aggressive treatment, does not agree to be transferred, or wants to be transferred to a specific area or specific hospital. Once a transfer request is received, an emergency physician from the NEMSR is provided with the demographic and clinical information in a physician-to-physician manner. Referrals are made in order of proximity, based on information in the NEMRIS and the geographic information system, which display hospitals that are available in close-range order. All requested patients are enrolled in the NEMSR registry. Hospital-, patient-, and time-related information are collected until the patient arrives at the receiving hospital.

### Study design and data collection

We conducted a retrospective, cross-sectional analysis using the NEMSR registry between 2017 and 2019. The following patients from the NEMSR registry were not included in this study: 1) patients for whom the referral request was withdrawn for any reason, 2) patients with missing age data, and 3) patients under 19 years of age.

We retrieved demographic and clinical information, including age, sex, the presence of a caregiver in the ED, requesting time, vital signs, the need for emergency surgery/procedure, primary diagnosis assigned using the Korean Classification of Diseases (KCD-7, a modified version of the 10th International Classification of Diseases), the number of hospitals contacted prior to the determination of transfer, the result of the transfer arrangement by the NEMSR, and disposition after transfer. We also extracted the reasons for transfer, the ED region (metropolitan city or province), the service level of the ED (level I ED or not), and the total beds in the sending hospital.

### Statistical analysis

The analysis was conducted to compare the characteristics of the patients with transfers arranged or not arranged by the NEMSR. Continuous variables with non-normal distribution are presented as medians (interquartile ranges), and categorical variables are presented as frequencies and proportions and tested by the Chi-squared test. We also determined the top 10 primary diagnoses of transferred patients. Hierarchical logistic regression models were constructed to investigate the independent predictors related to the transfer arrangements. Model 1 was constructed with patient-related variables, including age, sex, caregiver in the ED, requesting time, consciousness, systolic blood pressure, and the need for emergency surgery/procedure. In model 2, hospital-related variables were added to model 1, including the reasons for transfer, the region and service level of the ED, and the total beds in the sending hospital. All tests were two-tailed, and results with *p*-values of < 0.05 were considered statistically significant. All data preparation and statistical analyses were performed using SAS ver. 9.4 (SAS Institute Inc., Cary, NC, USA).

### Ethics

This study was approved by the Institutional Review Board of the National Medical Center (NMC-2021-12-143) and performed in accordance with the provisions of the Declaration of Helsinki. The requirement for obtaining informed consent from patients was waived by the board due to the observational nature of the study.

## Results

### Outcome of NEMSR operation

From 2017 to 2019, there were 24,621,061 ED visits by adult patients across the country [6]. Although the number of transferred patients between EDs was not published before 2019, about 1.6% of patients visited ED was known to be transferred to other ED according to the annual report after 2020 [9]. By applying the same proportion, it was estimated that there were about 393,937 interhospital transfers in Korea between 2017 and 2019.

For the same period, the NEMSR received 14,003 requests for transfer arrangements from 427 EDs (Supplementary Fig. 2). Of the 10,222 requests included in the final analysis, the NEMSR successfully arranged 8297 (81.17%) transfers. Among 1925 (18.83%) cases that could not be arranged by the NEMSR, 1335 (13.06%) were finally transferred by the sending EDs and 590 (5.77%) cases were not identified regarding determination. For 9970 cases with available data, the median time taken to arrange transfer by the NEMSR was 25 minutes and the 75th percentile time was 44 minutes. The median number of total hospitals contacted by both the NEMSR and the sending ED was four per transfer coordination, and the maximum number was 45 hospitals in one case.

### Patient characteristics

Of the arrangement episodes, the median age of the patients to be transferred was 65 years. Of all patients, 74.37% were male, and 88.40% visited the ED with their caregivers (Table 1). More than 32% of the patients required emergency surgery or procedures. The most frequent diagnosis was peritonitis, followed by other emergency conditions, such as intracranial injury, subarachnoid hemorrhage, aortic aneurysm and dissection, and acute myocardial infarction (Table 2). The most common reasons for transfer were the shortage of medical staff (59.79%) and ICU beds (30.80%) (Table 1). Requests from the EDs of hospitals with fewer than 600 beds comprised 55.65% of the total requests. Patients with transfers arranged by the NEMSR were most frequently sent to receiving hospitals with 600–799 beds.

**Table 1:** Patient and hospital characteristics of the cases requesting transfer arrangements

Characteristics	Number of patients (%)
Sex (*n* = 8288)	
Male	6164 (74.37)
Female	2124 (25.63)
Age (*n* = 10,222)	
19–44 yr	1420 (13.89)
45–59 yr	2503 (24.49)
60–69 yr	1959 (19.16)
70–79 yr	2273 (22.24)
≥ 80 yr	2067 (20.22)
Caregiver in ED (*n* = 10,131)	
Absent	1175 (11.60)
Present	8956 (88.40)
Requesting time (*n* = 9995)	
9 am–6 pm	3924 (39.26)
6 pm–9 am	6071 (60.74)
Consciousness level (*n* = 9417)	
Alert	6562 (69.68)
Not alert (CVPU^a^)	2855 (30.32)
Systolic blood pressure, mmHg (*n* = 6205)	
111–219	3738 (60.24)
101–110	761 (12.26)
91–100	695 (11.20)
0–90 or ≥ 220	1011 (16.29)
Need for emergency surgery/procedure (*n* = 10,222)	
No	6949 (67.98)
Yes	3273 (32.02)
Reason for transfer (*n* = 10,222)	
Shortage of general beds	245 (2.40)
Shortage of ICU beds	3148 (30.80)
Shortage of isolation/psychiatric beds	299 (2.93)
Shortage of medical staff	6112 (59.79)
Shortage of medical equipment/facility	227 (2.22)
Need for special treatment^b^	90 (0.88)
Others	101 (0.99)
Region of sending hospital (*n* = 10,222)	
Metropolitan city	7594 (74.29)
Province	2628 (25.71)
Service level I ED^c^ (*n* = 10,222)	
No	8483 (82.99)
Yes	1739 (17.01)
Total beds in sending hospital (*n* = 10,025)	
< 300	2841 (28.34)
300–599	2738 (27.31)
600–799	1964 (19.59)
800–999	1338 (13.35)
≥ 1000	1144 (11.41)
Number of hospitals contacted prior to determination (*n* = 10,222)	
0–5	6285 (61.49)
≥ 6	3937 (38.51)
Result of arrangement by NEMSR (*n* = 10,222)	
Arranged	8297 (81.17)
Not arranged	1925 (18.83)
Region of receiving hospital (*n* = 6504)	
Metropolitan city	5454 (83.86)
Province	1050 (16.14)
Total beds in receiving hospital (*n* = 6460)	
< 300	555 (8.59)
300–599	1436 (22.23)
600–799	1777 (27.51)
800–999	1612 (24.95)
≥ 1000	1080 (16.72)
Disposition after transfer (*n* = 6526)	
Hospitalization	3950 (60.53)
Emergency room	2252 (34.51)
Re-transfer	106 (1.62)
Home	131 (2.01)
Others	87 (1.33)

^a^Confusion, Voice, Pain, and Unresponsive by the National Early Warning System (NEWS)

^b^hyperbaric oxygen therapy, treatment for burns, surgery for finger amputation, etc.

^c^designated by the Korean Ministry of Health and Welfare

**Table 2:** Top 10 most common diagnoses of the cases requesting transfer arrangements

Ranking	Diagnostic code^a^	Diagnosis	Number of cases (%)
1	K65	Peritonitis	669 (6.54)
2	S06	Intracranial injury	583 (5.70)
3	I60	Subarachnoid hemorrhage	445 (4.35)
4	I71	Aortic aneurysm and dissection	368 (3.60)
5	I21	Acute myocardial infarction	326 (3.19)
6	I61	Intracerebral hemorrhage	321 (3.14)
7	I62	Other nontraumatic intracranial hemorrhage	294(2.88)
8	J18	Pneumonia, organism unspecified	266 (2.60)
9	I46	Cardiac arrest	263 (2.57)
10	R57	Shock, not elsewhere classified	245 (2.40)

^a^Korean Classification of Diseases, seventh revision (KCD-7), which is a modified version of the International Classification of Diseases, the tenth revision (ICD-10)

**Fig. 2:**
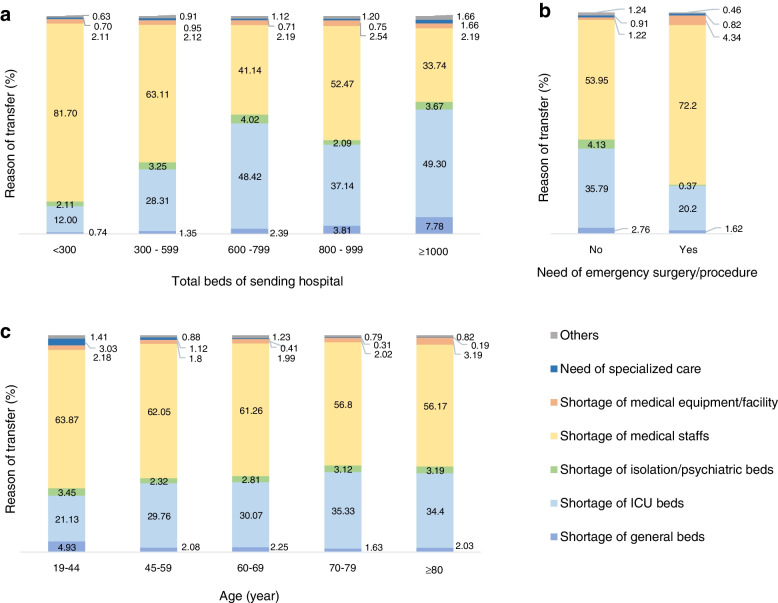
Distribution of reasons for transfer from emergency departments in Korea from 2017 to 2019. Proportion of transfer reasons presented by the total bed capacity of the sending hospital (**a**), for patients who needed emergency surgery or procedure (**b**), and the age of the patients who needed to be transferred (**c**)

As shown in Fig. 2a, the total number of hospital beds in sending EDs was closely associated with the reason for transfer. A shortage of ICU beds was the most common reason in hospitals with more than 1000 beds (49.30%) or 600–799 beds (48.42%). The more beds the sending hospital had, the greater the number of the transfer requests due to a shortage of ICU beds (*p* for trend < 0.0001). The smaller the bed capacity of the sending hospital, the more the transfer requests were related to a lack of medical staff. When a patient needed emergency surgery or procedure, most of the transfer requests were due to the shortage of medical staff (Fig. 2b). The older the patients who needed transfer, the higher the frequency of transfer requests due to a lack of ICU beds (Fig. 2c, *p* for trend < 0.0001).

### Factors associated with transfers that could not be arranged by the NEMSR

The proportion of could-not-be-arranged cases by the NEMSR increased slightly with increases in patient age, although such increases were not statistically significant (Table 3). With increasing patient age, transfer coordination was more likely to take longer than 44 minutes (Supplementary Table 5). Cases with caregivers in the ED were more successfully arranged for transfer than cases without caregivers (Table 3). Of the arrangement episodes, transfer requests due to the lack of isolation/closed beds had the lowest proportion of successful arrangements, followed by transfer requests due to a shortage of general beds. When transfers were requested due to a shortage of beds (including isolation/closed beds, general beds, or ICU beds), the proportion of arrangements taking more than 44 minutes was higher (Supplementary Table 5). The larger the sending hospital, the lower the proportion of successfully arranged transfers and the higher the proportion of arrangements that took longer (Table 3, Supplementary Table 5).

**Table 3:** Characteristics associated with interhospital transfer arrangements by the NEMSR

Characteristics	Arranged (%)	Could not be arranged (%)	*p*-value
Sex (*n* = 8288)			
Male	4992 (80.99)	1172 (19.01)	0.44
Female	1704 (80.23)	420 (19.77)	
Age (*n* = 10,222)			
19–44 yr	1174 (82.68)	246 (17.32)	0.33
45–59 yr	2023 (80.82)	480 (19.18)	
60–69 yr	1606 (81.98)	353 (18.02)	
70–79 yr	1836 (80.77)	437 (19.23)	
≥ 80 yr	1658 (80.77)	409 (19.79)	
Caregiver in ED (*n* = 10,131)			
Absent	891 (75.83)	284 (24.17)	<.0001
Present	7341 (81.97)	1615 (18.03)	
Requesting time (*n* = 9995)			
9 am–6 pm	3270 (83.33)	654 (16.67)	<.0001
6 pm–9 am	4840 (79.72)	1231(20.28)	
Consciousness level (*n* = 9417)			
Alert	5347 (81.48)	1215 (18.52)	0.56
Not alert (CVPU^a^)	2312 (80.98)	543 (19.02)	
Systolic blood pressure, mmHg (*n* = 6205)			
111–219	3100 (82.93)	638 (17.07)	0.26
101–110	630 (82.79)	131 (17.21)	
91–100	557 (80.14)	138 (19.86)	
0–90 or ≥ 220	822 (81.31)	189 (18.69)	
Need for emergency surgery/procedure (*n* = 10,222)			
No	5661 (81.46)	1288 (18.54)	0.26
Yes	2636 (80.54)	637 (19.46)	
Reason for transfer (*n* = 10,222)			
Shortage of general beds	184 (75.10)	61 (24.90)	<.0001
Shortage of ICU beds	2575(81.80)	573 (18.20)	
Shortage of isolation/psychiatric beds	208 (69.57)	91 (30.43)	
Shortage of medical staff	4995 (81.72)	1117 (18.28)	
Shortage of medical equipment/facility	184 (81.06)	43 (18.94)	
Need for special treatment^b^	80 (88.89)	10 (11.11)	
Others	71 (70.30)	30 (29.70)	
Region of sending hospital (*n* = 10,222)			
Metropolitan city	6104 (80.38)	1490 (19.62)	0.0005
Province	2193 (83.45)	435 (16.55)	
Service level I ED^c^ (*n* = 10,222)			
No	6920 (81.57)	1563 (18.43)	0.02
Yes	1377 (79.18)	362 (20.82)	
Total beds in sending hospital (*n* = 10,025)			
< 300	2403 (84.58)	438 (15.42)	<.0001
300–599	2234 (81.59)	504 (18.41)	
600–799	1576 (80.24)	388 (19.76)	
800–999	1052 (78.62)	286 (21.38)	
≥ 1000	857 (74.91)	287 (25.09)	

^a^Confusion, Voice, Pain, and Unresponsive by the National Early Warning System (NEWS)

^b^hyperbaric oxygen therapy, treatment for burns, surgery for finger amputation, etc.

^c^designated by the Korean Ministry of Health and Welfare

In multivariable analysis, the transfer of patients with caregivers in the ED was more likely to be successfully arranged (model 1, odds ratio [OR] for not arranged = 0.52, 95% confidence interval [CI]: 0.40–0.67; model 2, OR for not arranged = 0.50, 95% CI: 0.38–0.64), vs. patients without caregivers) (Table 4). However, transfer requests at nighttime (model 1, OR = 1.35, 95% CI: 1.15–1.58; model 2, OR = 1.33, 95% CI: 1.13–1.55, vs. daytime) and transfers requiring emergency surgery or procedures (model 1, OR = 1.22, 95% CI 1.04–1.43; model 2, OR = 1.28, 95% CI: 1.07–1.51, vs. not requiring) were less likely to be successfully arranged. Requests from the EDs of hospitals with more than 1000 beds had the lowest probability of successful arrangement (OR = 2.04, 95% CI: 1.48–2.82, ≥ 1000 beds vs. < 300 beds). Transfers requiring emergency surgery or procedures and transfers requested by the EDs of larger hospitals were more likely to take more than 44 minutes to arrange (Supplementary Table 6).

**Table 4:** Adjusted odds ratio for cases that could not be arranged by the NEMSR

Characteristics	Model 1 (*n* = 4671)		Model 2 (*n* = 4589)	
	aOR	*p*-value (95% CI)	aOR	*p*-value (95% CI)
Sex				
Male	1		1	
Female	1.07	0.43 (0.89–1.28)	1.07	0.45 (0.89–1.28)
Age				
19–44 yr	1		1	
45–59 yr	1.30	0.06 (0.98–1.71)	1.35	0.03 (1.01–1.79)
60–69 yr	1.24	0.15 (0.92–1.65)	1.24	0.16 (0.91–1.66)
70–79 yr	1.27	0.09 (0.95–1.68)	1.29	0.08 (0.96–1.71)
≥ 80 yr	1.38	0.031 (1.02–1.83)	1.44	0.01 (1.06–1.93)
Caregiver in ED				
Absent	1		1	
Present	0.52	<.0001 (0.40–0.67)	0.50	<.0001 (0.38–0.64)
Requesting time				
9 am–6 pm	1		1	
6 pm–9 am	1.35	0.0001 (1.15–1.58)	1.33	0.0005 (1.13–1.55)
Consciousness				
Alert	1		1	
Not alert (CVPU^a^)	0.98	0.81 (0.83–1.15)	1.02	0.82 (0.85–1.21)
Systolic BP				
111–219	1		1	
101–110	0.92	0.48 (0.72–1.16)	0.87	0.27 (0.68–1.11)
91–100	1.22	0.10 (0.96–1.54)	1.2	0.13 (0.94–1.52)
0–90 or ≥ 220	1.16	0.16 (0.94–1.42)	1.17	0.13 (0.95–1.44)
Need for emergency surgery/procedure				
No	1		1	
Yes	1.22	0.01 (1.04–1.43)	1.28	0.004 (1.07–1.51)
Reason for transfer				
Shortage of general beds			1	
Shortage of ICU beds			0.74	0.31 (0.42–1.31)
Shortage of isolation/psychiatric beds			1.87	0.07 (0.93–3.73)
Shortage of medical staff			0.81	0.47 (0.45–1.43)
Shortage of medical equipment/facility			0.55	0.10 (0.26–1.14)
Need for special treatment^b^			0.49	0.29 (0.12–1.86)
Others			1.27	0.65 (0.44–3.60)
Region of sending hospital				
Metropolitan city			1	
Province			1.01	0.94 (0.82–1.22)
Service level I ED^c^				
No			1	
Yes			0.95	0.71 (0.74–1.21)
Total beds in sending hospital				
< 300			1	
300–599			1.45	0.0007 (1.17–1.79)
600–799			1.37	0.01 (1.06–1.76)
800–999			1.46	0.01 (1.10–1.96)
≥ 1000			2.04	<.0001 (1.48–2.82)

*aOR* adjusted odds ratio, *CI* confidence interval

^a^Confusion, Voice, Pain, and Unresponsive by the National Early Warning System (NEWS)

^b^hyperbaric oxygen therapy, treatment for burns, surgery for finger amputation, etc.

^c^designated by the Korean Ministry of Health and Welfare

## Discussion

Similar to the NEMSR, regionalized emergency care systems for some defined emergencies, such as trauma [10], ST-elevation myocardial infarction [11], and stroke [12], require a central coordinating function to transfer patients to designated centers [5]. Compared to these coordinating services, coordination by the NEMSR is aimed at transfers not limited to a certain diagnosis or status, and thus, it may be more challenging to optimize the destination on occasion. The first key element to performing transfer coordination by the NEMSR is the protocolized transfer process and communication by public coordinators. Prior qualitative studies showed that transfer coordination is a contentious and time-consuming process [2, 3], and poor communication are known to be common in transfer arrangements within in ED [5, 13]. However, the transfer process and communication may determine the patient’s destination and the time it takes to coordinate the transfer. As shown in previous studies, a standardized process using protocols and effective communication is critical to streamlining the transfer process, and presents an opportunity to facilitate transfers [14, 15].

Another element is the use of a resource and geographic information system, namely, the NEMRIS, to determine referral hospitals in a short time. For emergency transfers, the coordinator needs to immediately identify referral hospitals with good geographical accessibility and sufficient medical resources. The NEMRIS plays a key role in enabling transfer arrangements by providing resource information in near-real time. During the COVID-19 pandemic, the use of the NEMRIS proved invaluable in the interhospital transfers of critically ill patients with COVID-19. Nevertheless, some improvements have been suggested to more actively utilize a resource availability system such as the NEMRIS. First, input mechanisms need to be devised to accurately and immediately reflect resource statuses, preferably in real-time. Some resource information might be automatically collected from the electronic medical information system of the hospital by the resource availability system. If the information manager is properly motivated, information on the availability of some emergency procedures or surgeries (e.g., reperfusion interventions for myocardial infarction and acute abdominal surgery) in the hospital can be relatively accurate. However, some information such as the ICU capacity is inherently context-dependent, as in case of when there are inpatients waiting for an ICU in the hospital. Even if it is provided in real-time, some information may still be inaccurate and require manual verification. Second, it was recently argued that the information system should include quality indicators of the hospital, such as clinical outcomes and teaching status for optimizing transfer processes [2, 3, 16, 17]. It is common to decide to transfer a patient to a hospital that can respond most quickly because there are few candidate hospitals that can receive emergency patients or the transfer arrangement itself is cumbersome [4]. Nevertheless, efforts to make a qualitatively informed decision for selecting the destination should continue, which can improve patient outcomes after transfer.

Current results identified that the shortage of the hospital’s resource by identifying the main reason for transfer. Smaller hospitals are impacted more by a shortage of medical staff than larger hospitals. Patients in smaller hospitals had to be transferred due to the lack of medical staff or a surgeon, particularly when emergency surgery or procedures were needed. Prior studies revealed that shortages of physicians and surgeons have steadily worsened in rural areas [18–20], leading to a lack of basic surgical access in small community hospitals [19]. While the service quality or resource availability of community hospitals may vary from case to case, supporting and improving the service level of small hospitals is a common challenge in many countries [1].

In contrast, there is widespread awareness that tertiary hospitals lack ICU beds [4]. The results of this study supported this perception, which showed that larger hospitals needed to transfer patients due to a lack of ICU beds. Notably, the older the patients, the more transfers were requested due to a lack of ICU beds. This is consistent with previous findings from the US showing that annual ICU admissions from EDs increased overall by 79% between 2001 and 2009, while the ICU admissions of patients 65 years and older increased by 131.3% [21, 22]. With the rapidly aging population, studies have predicted that ICU demand and the bed occupancy rate will increase by varying degrees [23, 24]. Additional studies are needed to estimate the true extent of ICU demand, considering the efficiency of ICU use and the operation of “drain beds” for critically ill but stable patients [4].

The issue to be briefly addressed pertains to the workforce of the transfer coordination team. The NEMSR has one or more emergency physician who supervise the transfer process, with two non-physician coordinators per duty. Since emergency physicians are scarce resources and the training of residents typically occurs on a small scale, non-physician coordinators can be appropriately trained and put into practice, as in prior situations [25, 26]. Well-trained and qualified coordinators increase the efficiency of the transfer process and perform their roles well where physicians are not available [27].

This study had several limitations inherent to the data. Since the NEMSR registry only included cases that requested transfer coordination by the NEMSR, the samples were not representative of all emergency interhospital transfers, which limited a comprehensive understanding of the transfer patterns. Referring to the recently published results [9], the number of transfer cases with the NEMSR’s coordination was likely to be less than 4.44% of all transfers for critically ill adults from 2017 to 2019. This was probably because the NEMSR’s service is not yet well known to EDs, or because EDs called for the NEMSR’s help only for a small number of cases where a transfer was not feasible. Secondly, this study was not able to assess the entire transfer process in detail, including processes before and after the NEMSR’s coordination. Data on the processes and communication in the sending EDs before the request to the NEMSR, more specific reasons for transfer from sending hospitals, and the treatment processes after transfer to the referral hospitals can reveal more reasons for delays. Finally, the patient’s final disposition and clinical outcome could not be identified for cases in which neither the NEMSR and the ED could determine the destination. Further comparative analysis of the process and results between all transfers and transfers coordinated by the NEMSR will provide a clearer picture of the effectiveness of the NEMSR operation.

## Conclusions

With the trend in increasing ED visits by critically ill patients for hospitalization, the operation of the NEMSR has great clinical importance in that it coordinates the interhospital transfers of emergency patients across the country based on a protocolized process and resource availability system. This study revealed that the main reason for the transfers was scarce hospital resources, and the type of scarce resource depended on the bed capacity of the sending hospitals. The role of the coordinator is even more significant as technology in emergency care develops and regional gaps in the distribution of medical resources widen.

## Supplementary Information

**Additional file 1: Supplementary Fig. 1.** The distribution of the designated EDs by the government in Korea. **Supplementary Fig. 2.** The flow diagram selecting study cases included in the final anaysis.

**Additional file 2: Supplementary Table 1.** The distribution of hospitals by size in Korea, 2019. **Supplementary Table 2.** Population, area, number of hospitals^*^ by Metropolitan City/Province in Korea, 2019. **Supplementary Table 3.** Emergency department (ED) occupancy rate (level I, II)^*^ by metropolitan City/province in Korea, 2019. **Supplementary Table 4.** Emergency department (ED) occupancy rate (level I, II)* by hospital type/size in Korea, 2019. **Supplementary Table 5.** Characteristics associated with time taken to arrange for interhospital transfer. **Supplementary Table 6.** Adjusted odds of for time taken to arrange ≥44 min by patient and hospital characteristics.

## Data Availability

The sharing of data from this study is restricted due to ethical and legal constrictions. The data contain sensitive personal health information, which is protected under The Personal Information Protection Act in Korea, thus making all data requests subject to Institutional Review Board (IRB) approval. Per National Medical Center (NMC) IRB, the data that support the findings of this study are restricted for transmission to those in the primary investigative team. Data sharing with investigators outside the team requires IRB approval. In this case, please contact Dr. Eunsil Ko, the corresponding author of this study. All requests for anonymized data will be reviewed by the research team and then submitted to the NMC IRB for approval.

## Abbreviations

ED: Emergency department
NEMSR: National Emergency Medical Situation Room
NEMRIS: National Emergency Medical Resource Information System
EMS: Emergency medical service
KCD-7: Korean Classification of Diseases 7th Edition
ICD-10: International Classification of Disease 10th Edition
ICU: Intensive care unit
OR: Odds ratio
CI: Confidence interval
