# Supplementary material for: Operation of national coordinating service for interhospital transfer from emergency departments: experience and implications from Korea
Source: BMC Emerg Med. 2023 Feb 10;23:15. doi: 10.1186/s12873-023-00782-1 (PMC9913013; doi:10.1186/s12873-023-00782-1)
Supplement: Supplementary file 1 — Additional file 1: Supplementary Fig. 1. The distribution of the designated EDs by the government in Korea. Supplementary Fig. 2. The flow diagram selecting study cases included in the final anaysis. [file 12873_2023_782_MOESM1_ESM.pptx]

## Slide 1
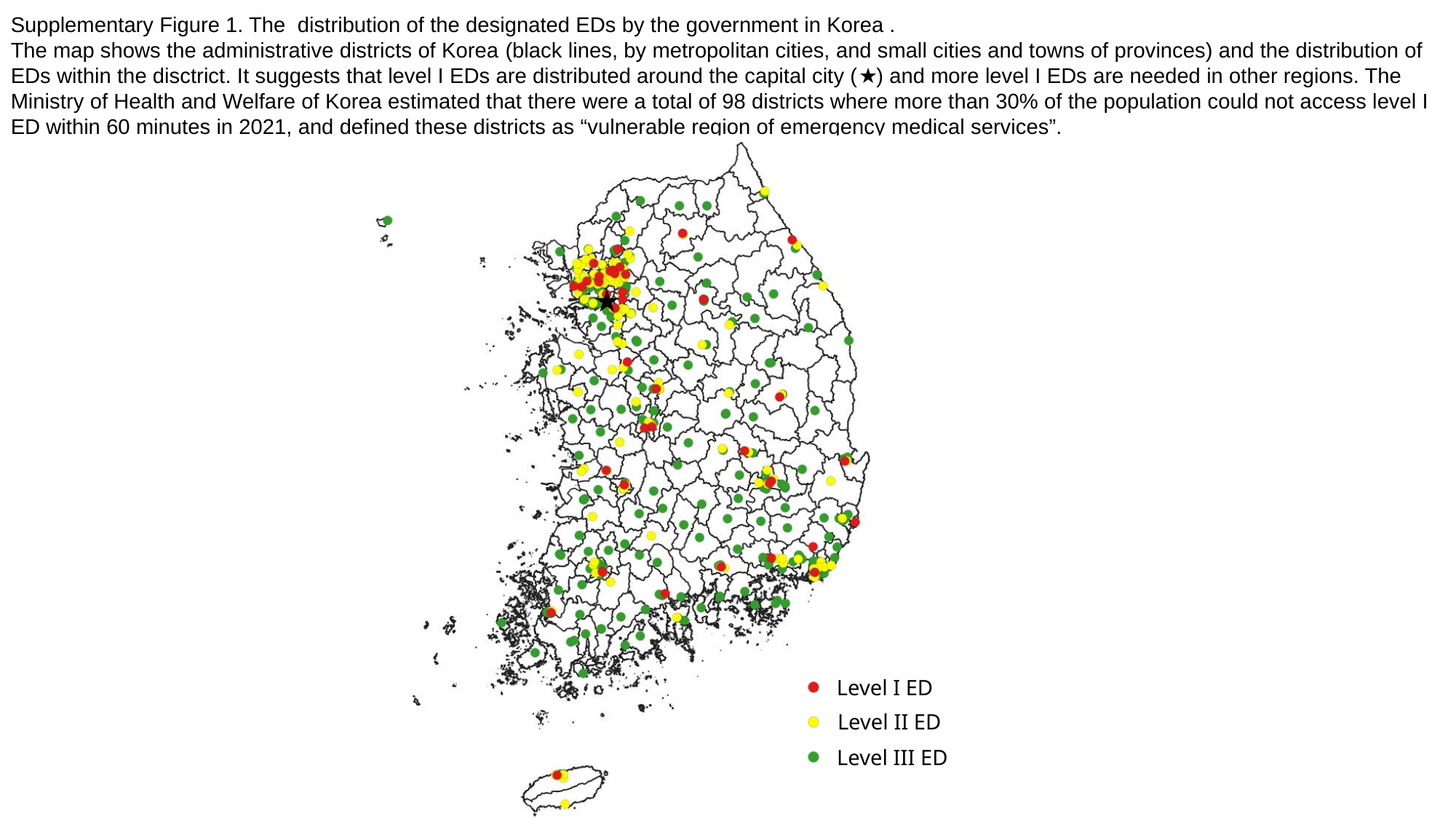

Supplementary Figure 1. The distribution of the designated EDs by the government in Korea .
The map shows the administrative districts of Korea (black lines, by metropolitan cities, and small cities and towns of provinces) and the distribution of EDs within the disctrict. It suggests that level I EDs are distributed around the capital city (★) and more level I EDs are needed in other regions. The Ministry of Health and Welfare of Korea estimated that there were a total of 98 districts where more than 30% of the population could not access level I ED within 60 minutes in 2021, and defined these districts as “vulnerable region of emergency medical services”.
★
Level I ED
Level II ED
Level III ED

## Slide 2
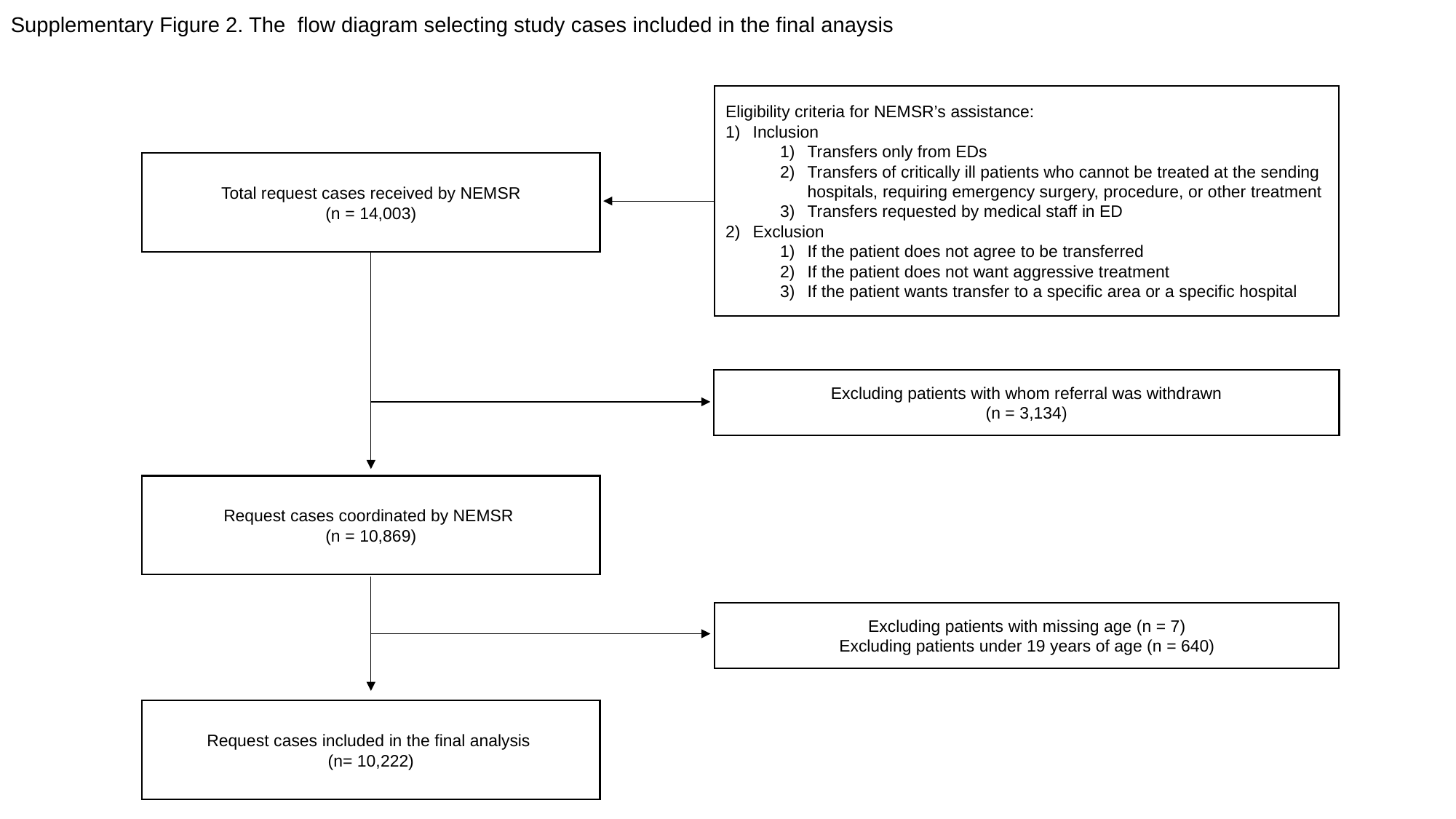

Supplementary Figure 2. The flow diagram selecting study cases included in the final anaysis
Eligibility criteria for NEMSR’s assistance:
Inclusion
Transfers only from EDs
Transfers of critically ill patients who cannot be treated at the sending hospitals, requiring emergency surgery, procedure, or other treatment
Transfers requested by medical staff in ED
Exclusion
If the patient does not agree to be transferred
If the patient does not want aggressive treatment
If the patient wants transfer to a specific area or a specific hospital
Total request cases received by NEMSR
(n = 14,003)
Excluding patients with whom referral was withdrawn
(n = 3,134)
Request cases coordinated by NEMSR
(n = 10,869)
Excluding patients with missing age (n = 7)
Excluding patients under 19 years of age (n = 640)
Request cases included in the final analysis
(n= 10,222)
