# Supplementary material for: Operation of national coordinating service for interhospital transfer from emergency departments: experience and implications from Korea
Source: BMC Emerg Med. 2023 Feb 10;23:15. doi: 10.1186/s12873-023-00782-1 (PMC9913013; doi:10.1186/s12873-023-00782-1)
Supplement: Supplementary file 2 — Additional file 2: Supplementary Table 1. The distribution of hospitals by size in Korea, 2019. Supplementary Table 2. Population, area, number of hospitals* by Metropolitan City/Province in Korea, 2019. Supplementary Table 3. Emergency department (ED) occupancy rate (level I, II)* by metropolitan City/province in Korea, 2019. Supplementary Table 4. Emergency department (ED) occupancy rate (level I, II)* by hospital type/size in Korea, 2019. Supplementary Table 5. Characteristics associated with time taken to arrange for interhospital transfer. Supplementary Table 6. Adjusted odds of for time taken to arrange ≥44 min by patient and hospital characteristics. [file 12873_2023_782_MOESM2_ESM.docx]

Supplementary Table 1. The distribution of hospitals by size in Korea, 2019

|  | Hospital size by number of beds | | | | | |
| --- | --- | --- | --- | --- | --- | --- |
|  | < 300 | 300–599 | 600–799 | 800–999 | ≥1000 | Total |
| Hospitals (No.) | 230 | 97 | 26 | 33 | 16 | 402 |
| Proportion | 57.2% | 24.1% | 6.5% | 8.2% | 4.0% | 100.0% |

Data source: 2020 Statistical Yearbook from the Ministry of the Interior and Safety, Korea; 2020 Cadastral Statistical Annual Report from the Ministry of Land, Infrastructure and Transport, Korea; National Emergency Medical Resource Information System (NEMRIS)

Supplementary Table 2. Population, area, number of hospitals^*^ by Metropolitan City/Province in Korea, 2019

| City/Province | Population | Land area(㎢) | No. of hospitals | No. of hospitals (≥500 beds) | No. of hospitals per ㎢ | No. of hospitals per 1,000 persons | No. of total beds | No. of beds per 1,000 persons |
| --- | --- | --- | --- | --- | --- | --- | --- | --- |
| Total | 51,849,861 | 100,401.3 | 402 | 94 | 0.0040 | 0.0078 | 157,552 | 3.0386 |
| Seoul | 9,729,107 | 605.2 | 50 | 24 | 0.0826 | 0.0051 | 32,647 | 3.3556 |
| Busan | 3,413,841 | 770.1 | 28 | 6 | 0.0364 | 0.0082 | 12,659 | 3.7081 |
| Daegu | 2,438,031 | 883.5 | 15 | 6 | 0.0170 | 0.0062 | 7,273 | 2.9831 |
| Incheon | 2,957,026 | 1,063.3 | 20 | 4 | 0.0188 | 0.0068 | 8,224 | 2.7812 |
| Gwangju | 1,456,468 | 501.1 | 20 | 4 | 0.0399 | 0.0137 | 6,847 | 4.7011 |
| Daejeon | 1,474,870 | 539.6 | 10 | 4 | 0.0185 | 0.0068 | 5,318 | 3.6057 |
| Ulsan | 1,148,019 | 1,062.0 | 8 | 2 | 0.0075 | 0.0070 | 3,064 | 2.6689 |
| Sejong^**^ | 340,575 | 464.9 | 1 | - | 0.0022 | 0.0029 | 200 | 0.5872 |
| Gyeonggi-do | 13,239,666 | 10,192.5 | 65 | 18 | 0.0064 | 0.0049 | 25,635 | 1.9362 |
| Gangwon-do | 1,541,502 | 16,828.3 | 22 | 3 | 0.0013 | 0.0143 | 6,330 | 4.1064 |
| Chungcheongbuk-do | 1,600,007 | 7,406.8 | 16 | 2 | 0.0022 | 0.0100 | 5,243 | 3.2769 |
| Chungcheongnam-do | 2,123,709 | 8,245.5 | 16 | 3 | 0.0019 | 0.0075 | 5,110 | 2.4062 |
| Jeollabuk-do | 1,818,917 | 8,069.1 | 20 | 4 | 0.0025 | 0.0110 | 6,178 | 3.3965 |
| Jeollanam-do | 1,868,745 | 12,345.2 | 38 | 3 | 0.0031 | 0.0203 | 10,350 | 5.5385 |
| Gyeongsangbuk-do | 2,665,836 | 19,033.3 | 31 | 5 | 0.0016 | 0.0116 | 9,142 | 3.4293 |
| Gyeongsangnam-do | 3,362,553 | 10,540.4 | 36 | 4 | 0.0034 | 0.0107 | 11,107 | 3.3031 |
| Jeju | 670,989 | 1,850.2 | 6 | 2 | 0.0032 | 0.0089 | 2,225 | 3.3160 |

^*^ The number of hospitals counts only hospitals with level I, II, and III emergency departments.

^**^ Sejong city is a special autonomic city with central government organizations, not a metropolitan city.

Data source: 2020 Statistical Yearbook from the Ministry of the Interior and Safety, Korea; 2020 Cadastral Statistical Annual Report from the Ministry of Land, Infrastructure and Transport, Korea; National Emergency Medical Resource Information System (NEMRIS)

Supplementary Table 3. Emergency department (ED) occupancy rate (level I, II)^*^ by metropolitan City/province in Korea, 2019

| City/Province | No. of EDs (Level I, II) | No. of ED beds (Level I, II) | No.of ED visits | ED occupancy rate (%) |
| --- | --- | --- | --- | --- |
| Total | 146 | 4,202 | 4,095,351 | 51.8 |
| Seoul | 30 | 1,013 | 1,030,612 | 55.4 |
| Busan | 6 | 168 | 127,886 | 60.6 |
| Daegu | 6 | 197 | 177,748 | 95.6 |
| Incheon | 9 | 240 | 273,497 | 35.9 |
| Gwangju | 4 | 118 | 102,711 | 62.3 |
| Daejeon | 5 | 149 | 152,652 | 48.3 |
| Ulsan | 2 | 65 | 61,011 | 39.9 |
| Sejong^**^ | - | - | - | - |
| Gyeonggi-do | 31 | 914 | 1,010,124 | 41.6 |
| Gangwon-do | 7 | 192 | 150,120 | 29.9 |
| Chungcheongbuk-do | 4 | 105 | 89,757 | 31.4 |
| Chungcheongnam-do | 8 | 194 | 203,519 | 29.3 |
| Jeollabuk-do | 7 | 172 | 139,609 | 43.3 |
| Jeollanam-do | 5 | 116 | 85,396 | 41.6 |
| Gyeongsangbuk-do | 9 | 212 | 186,847 | 25.6 |
| Gyeongsangnam-do | 8 | 231 | 182,541 | 33.9 |
| Jeju | 5 | 116 | 121,321 | 32.1 |

^*^ ED occupancy rate = (Number of patient × length of stay in ED as mean time, hour) / (Number of beds × number of days × 24 hours)

^**^ Sejong city is a special autonomic city with central government organizations, not a metropolitan city

Data source: Evaluation Result Report of Emergency Department from National Emergency Medical Center in Korea, 2019

Supplementary Table 4. Emergency department (ED) occupancy rate (level I, II)^*^ by hospital type/size in Korea, 2019

| Hospital type/size | | No. of EDs | No. of ED beds | No. of ED visits | ED occupancy rate (%) |
| --- | --- | --- | --- | --- | --- |
| Total | | 146 | 4,202 | 4,095,351 | 51.8 |
| Tertiary hospital^**^ | | 39 | 1,489 | 1,532,700 | 84.1 |
| General hospital^***^ | ≥ 300 | 78 | 2,103 | 2,088,621 | 38.0 |
|  | < 300 | 29 | 610 | 474,030 | 18.3 |

^*^ ED occupancy rate = (Number of patient × length of stay in ED as mean time, hour) / (Number of beds × number of days × 24 hours)

^**^ More than 500 beds and large-sized hospitals designated by the Korean Ministry of Health and Welfare; ^***^ More than 100 beds with several specialty departments designated by law

Data source: Evaluation Result Report of Emergency Department from National Emergency Medical Center in Korea, 2019

Supplementary Table 5. Characteristics associated with time taken to arrange for interhospital transfer

| **Characteristics** | | **Time taken to arrange**  **< 44 min (%)** | **Time taken to arrange**  **≥ 44 min (%)** | **p value** |
| --- | --- | --- | --- | --- |
| Sex (n=8,119) | Male | 4,531(75.25) | 1,490(24.75) | 0.0004 |
|  | Female | 1,496(71.31) | 602(28.69) |  |
| Age (n=9,970) | 19-44yr | 1,069(77.35) | 313(22.65) | 0.009 |
|  | 45-59yr | 1,850(75.91) | 587(24.09) |  |
|  | 60-69yr | 1,423(74.70) | 482(25.30) |  |
|  | 70-79yr | 1,637(73.77) | 582(26.23) |  |
|  | ≥ 80yr | 1,469(72.47) | 558(27.53) |  |
| Caregiver in ED (n=9,880) | Absence | 843(76.22) | 263(23.78) | 0.22 |
|  | Presence | 6,537(74.50) | 2,237(25.50) |  |
| Requesting time (n=9,970) | 9am-6pm | 2,945(75.28) | 967(24.72) | 0.29 |
|  | 6pm-9am | 4,503(74.33) | 1,555(25.67) |  |
| Consciousness (n=9,167) | Alert | 4,734(74.22) | 1,644(25.78) | 0.39 |
|  | Not alert (CVPU*) | 2,094(75.08) | 695(24.92) |  |
| Systolic BP (n=6,086) | 1: 111 - 219 | 2,713(74.15) | 946(25.85) | 0.0005 |
|  | 2: 101 - 110 | 532(71.03) | 217(28.97) |  |
|  | 3: 91 - 100 | 462(68.04) | 217(31.96) |  |
|  | 4: 0 - 90, ≥ 220 | 763(76.38) | 236(23.62) |  |
| Need of emergency surgery/procedure (n=9,970) | No | 5,087(75.79) | 1,625(24.21) | 0.0003 |
|  | Yes | 2,361(72.47) | 897(27.53) |  |
| Reasons for transfer (n=9,970) | Shortage of general beds | 164(68.91) | 74(31.09) | <.0001 |
|  | Shortage of ICU beds | 2,181(70.77) | 901(29.23) |  |
|  | Shortage of isolation/psychiatric beds | 179(60.88) | 115(39.12) |  |
|  | Shortage of medical staffs | 4,597(77.25) | 1,354(22.75) |  |
|  | Shortage of medical equipment/facility | 169(74.78) | 57(25.22) |  |
|  | Need of special treatment** | 76(92.68) | 6(7.32) |  |
|  | Others | 82(84.54) | 15(15.46) |  |
| Region of sending hospital (n=10,222) | Metropolitan city | 5,355 (72.11) | 2,071 (27.89) | <.0001 |
|  | Province | 2,093 (82.27) | 451 (17.73) |  |
| Service level I ED *** (n=9,970) | No | 6,307(76.08) | 1,983(23.92) | <.0001 |
|  | Yes | 1,141(67.92) | 539(32.08) |  |
| Total beds of sending hospital (n=9,782) | < 300 | 2,338(83.95) | 447(16.05) | <.0001 |
|  | 300 - 599 | 2,020(75.94) | 640(24.06) |  |
|  | 600 - 799 | 1,357(70.60) | 565(29.40) |  |
|  | 800 - 999 | 883(67.15) | 432(32.85) |  |
|  | ≥ 1000 | 688(62.55) | 412(37.45) |  |

*Confusion; Voice; Pain; Unresponsive, by the National Early Warning System (NEWS)

**Hyperbaric oxygen therapy, treatment for burn, surgery for finger amputation, etc.

*** Designated by the Korean Ministry of Health and Welfare

Supplementary Table 6. Adjusted odds of for time taken to arrange ≥ 44 min by patient and hospital characteristics

| **Characteristics** | | **Model 1 (n=4,666)** | | **Model 2 (n=4,584)** | |
| --- | --- | --- | --- | --- | --- |
|  |  | **aOR^a^** | **p value**  **(95% CI)** | **aOR** | **p value**  **(95% CI)** |
| Sex | Male | 1 |  | 1 |  |
|  | Female | 1.06 | 0.47  (0.89-1.28) | 1.06 | 0.43  (0.91-1.24) |
| Age | 19-44yr | 1 |  | 1 |  |
|  | 45-59yr | 1.03 | 0.75  (0.82-1.31) | 1.01 | 0.94  (0.79-1.28) |
|  | 60-69yr | 1.17 | 0.21  (0.91-1.49) | 1.08 | 0.50  (0.84-1.39) |
|  | 70-79yr | 1.13 | 0.31  (0.89-1.43) | 1.03 | 0.78  (0.81-1.32) |
|  | ≥ 80yr | 1.33 | 0.02  (1.05-1.27) | 1.26 | 0.06  (0.98-1.62) |
| Caregiver in ED | Absence | 1 |  | 1 |  |
|  | Presence | 0.89 | 0.37  (0.69-0.15) | 0.81 | 0.10  (0.62-1.04) |
| Requesting time | 9am-6pm | 1 |  | 1 |  |
|  | 6pm-9am | 1.14 | 0.05  (0.99-1.30) | 1.06 | 0.39  (0.92-1.21) |
| Consciousness | Alert | 1 |  | 1 |  |
|  | Not alert (CVPU*) | 0.99 | 0.94  (0.86-1.15) | 0.95 | 0.55  (0.82-1.11) |
| Systolic BP | 1: 111 - 219 | 1 |  | 1 |  |
|  | 2: 101 - 110 | 1.16 | 0.12  (0.95-1.42) | 1.08 | 0.43  (0.88-1.32) |
|  | 3: 91 - 100 | 1.33 | 0.006  (1.08-1.63) | 1.28 | 0.01  (1.04-1.58) |
|  | 4: 0 - 90, ≥ 220 | 0.92 | 0.36  (0.76-1.11) | 0.93 | 0.45  (0.76-1.12) |
| Need of emergency surgery/procedure | No | 1 |  | 1 |  |
|  | Yes | 1.25 | 0.001  (1.08-1.43) | 1.37 | <.0001  (1.18-1.59) |
| Reasons for transfer | Shortage of general beds |  |  | 1 |  |
|  | Shortage of ICU beds |  |  | 1.65 | 0.06  (0.96-2.85) |
|  | Shortage of isolation/psychiatric beds |  |  | 2.86 | 0.001  (1.48-5.52) |
|  | Shortage of medical staffs |  |  | 1.27 | 0.38  (0.73-2.19) |
|  | Shortage of medical equipment/facility |  |  | 1.27 | 0.47  (0.66-2.44) |
|  | Need of special treatment** |  |  | <0.001 | 0.96  (<0.001->999.) |
|  | Others |  |  | 1.15 | 0.79  (0.39-3.35) |
| Region of sending hospital (n=10,222) | Metropolitan city |  |  | 1 |  |
|  | Province |  |  | 0.74 | 0.001  (0.61-0.88) |
| Service level I ED *** | No |  |  | 1 |  |
|  | Yes |  |  | 1.05 | 0.61  (0.85-1.30) |
| Total beds of sending hospital | < 300 |  |  |  |  |
|  | 300 - 599 |  |  | 1.44 | 0.0002  (1.19-1.75) |
|  | 600 - 799 |  |  | 1.74 | <.0001  (1.40-2.16) |
|  | 800 - 999 |  |  | 1.95 | <.0001  (1.52-2.50) |
|  | ≥ 1000 |  |  | 2.08 | <.0001  (1.57-2.76) |

^a^ Adjusted Odds Ratio

*Confusion; Voice; Pain; Unresponsive, by the National Early Warning System (NEWS)

**Hyperbaric oxygen therapy, treatment for burn, surgery for finger amputation, etc.

*** Designated by the Korean Ministry of Health and Welfare
